# Flow and Meaningfulness as Mechanisms of Change in Self-Concept and Well-Being Following a Songwriting Intervention for People in the Early Phase of Neurorehabilitation

**DOI:** 10.3389/fnhum.2015.00299

**Published:** 2015-05-26

**Authors:** Felicity Anne Baker, Nikki Rickard, Jeanette Tamplin, Chantal Roddy

**Affiliations:** ^1^The University of Melbourne, Melbourne, VIC, Australia; ^2^Monash University, Melbourne, VIC, Australia

**Keywords:** songwriting, self-concept clarity, spinal cord injuries, acquired brain injury, depression and anxiety disorders, well-being, flow theory

## Abstract

Anecdotal evidence suggests that songwriting assists people with spinal cord injury (SCI) or acquired brain injury (ABI) to explore threats to self-concept, yet studies that explore the mechanisms of change have not been reported. In a pilot study, we explored the correlations between changes in self-concept and well-being, with mechanisms of flow and meaningfulness of songwriting. Five people with ABI (all male) and 5 SCI (4 males, 1 female) (mean age 38.90 years, SD = 13.21), with an average 3 months post-injury, participated in a 12-session songwriting program that targeted examination of self-concept. Measures of self-concept, depression, anxiety, emotion regulation, affect, satisfaction with life, and flourishing were collected pre-, mid-, and post-intervention, and compared with repeated measures of flow and meaningfulness of songwriting. Medium effects were found for changes in self-concept (*d* = 0.557) and depression (*d* = 0.682) and approached a medium effect for negative affect (*d* = 0.491). Improvements in self-concept over time were associated with decreases in depression (*r*_p_ = −0.874, *n* = 9, *p* < 0.01), anxiety (*r*_p_ = −0.866, *n* = 9, *p* < 0.01), and negative affect (*r*_p_ = −0.694, *n* = 10, *p* < 0.05), and an increase in flourishing (*r*_p_ = +0.866, *n* = 9, *p* < 0.01) and positive affect (*r*_p_ = + 0.731, *n* = 10, *p* < 0.05). Strong experiences of flow were not positively correlated with positive changes to self-concept and well-being, whereas deriving high levels of meaning were associated with increased negative affect (*r*_p_ = +0.68 *p* < 0.05), increased anxiety (*r*_p_ = +0.74, *p* < 0.05), and reduced emotional suppression (*r*_p_ = −0.58, *p* < 0.05). These findings show that the targeted songwriting intervention appears to be positively associated with enhanced well-being outcomes. However, the findings also suggest that people who find the songwriting process has strong meaning for them might be more likely to start accepting their emotions and as a result experience an increase in anxiety and depression, although full, mediated regression analyses with larger sample sizes are required to explore this further. Acknowledging their changed circumstances may nonetheless assist people with SCI and ABI to grieve their losses and facilitate the building of a healthy post-injured self-concept. We propose that there may be other mechanisms more critical in facilitating the positive changes in self-concept and well-being than flow and meaning, such as the role of story-telling and the impact of music in facilitating the consolidation of self-concept explorations in memory.

## Introduction

The self-concept is a set of beliefs that, when combined, enables people to have a sense of who they are in the world. The self-concept is derived from an integration of self-schemas constructed by temporal frameworks that encompass the past, present, and future selves (Markus and Nurius, 1986) and is multidimensional, comprising the personal, physical, family, social, academic/vocational, and spiritual/moral self domains (Fitts and Warren, 1996). Recent research suggests that the self-concept has neuropsychological and structural properties that, when combined, form a continuum from unhealthy-fragmented to healthy-integrated self-concept (Gana, 2012). Importantly, the self-concept forms a nomological network of relationships that involve an individual’s personality, psychological adjustment, and achievement (Gana, 2012). Emotional or significant life events may be catalysts for personal examination of the self-concept, particularly when these stimulate opportunities to reassess purpose and meaning in life and construct an alternative future self that aligns with realistic possibilities (Habermas and Bluck, 2000). People who report having a strong sense of self tend to thrive because encountering failures in life does not dramatically affect their positive view of themselves. However, for those with a more negative self-concept, there is a risk that they will not achieve in life because they tend to avoid opportunities for growth by avoiding risk (Fitts and Warren, 1996).

The concept of the self is constantly evolving throughout life as a consequence of maturation and encountering new people and experiences (MacKinnon and Helse, 2010). However, in some circumstances, a significant and traumatic life event interrupts this gradual process and demands a more focused review of the past, present, and future selves. Acquiring a neurodisability calls for a reappraisal of the self and may involve a process of grieving for components of the self that have been damaged or lost as a result of the trauma (Hinkebein and Stucky, 2007). Finding meaning, purpose, and fulfillment in life is challenging for people with acquired neurodisabilities (Vickery et al., 2005) and there is a risk for people with acquired brain injury (ABI) or spinal cord injury (SCI) that the lens through which they frame and experience life will be dominated by the “disabled self” if an integrated and balanced self-concept is not constructed post-injury (Lennon et al., 2014). A number of studies have concluded that people with ABI or SCI report incongruities in past, present, and future selves that do not improve naturally over time when compared to the normal population (Anson and Ponsford, 2006; Kelly et al., 2013).

Engaging people with ABI or SCI in a narrative process that explores the residual self alongside the disabled self enables them to grieve the lost self and construct a new and healthy present and future self (Feinstein and Krippner, 2008). When provided with opportunities to tell and retell their stories, and have a listener support and gently challenge their perceptions of themselves, alternative selves are identified and long-term integration of the self-concept is more likely (Obodaru, 2012).

Therapeutic songwriting is a method that has been extensively used across a range of clinical and non-clinical populations as a medium for people to tell their stories (Baker et al., 2008). In a study of song lyrics created by people with ABI, inductively derived themes illustrated that their songs focused on past, present, and future selves (Baker et al., 2005a,b,c). Songs about the past included descriptions of relationships with significant others and past events (16.9%). Songs about the present comprised reflections on or sending messages to family and friends (22.6%) or expressions of their adversity in relation to their physical impairments and their efforts in rehabilitation (9.6%). Of particular interest, 28% of the lyrics focused on self-reflections, including questioning life’s meaning and describing what makes them happy. A small percentage of the lyrics (7.4%) also focused on the future self. The songs reviewed in this study were, however, not created according to any specific protocol, as they were drawn from the large collection of songs that had been accumulated over a number of years of therapeutic practice and later analysed. We have recently developed and piloted a songwriting protocol specifically designed to explore the past, present, and future self for people with ABI or SCI using a narrative approach (Tamplin et al., 2015). Constructing the most effective protocol is, however, also likely to be dependent upon understanding the mechanisms of change. Currently, no songwriting study has specifically tested which mechanisms are active during the songwriting process.

### Theory of mechanisms of change

In an earlier article (Tamplin et al., 2015), we presented a theory of possible mechanisms active during the songwriting process for people with neurodisability that enabled them to successfully integrate multiple injured and non-injured narratives. We proposed that songwriting accommodates for memory impairments typical in people with ABI because of the strong links between music, memory, and emotions, which enable exploration of the self to be consolidated more effectively in memory (Cahill and McGaugh, 1996; Judde and Rickard, 2010) and stimulate autobiographical memories that are important in raising awareness of the residual self (Janata, 2009). Our mechanisms of change theory suggests that because engagement in music-based activities activates the neural “pleasure” network in the brain (e.g., Menon and Levitin, 2005; Salimpoor and Zatorre, 2013), songwriting has the potential to enhance mood and coping and decrease or prevent depression and anxiety. Through achieving this positive-affect shift, people may access the inner strength needed to face the challenges associated with processing and revising their self-concept post-injury.

Flow theory is of particular importance in our theory of mechanisms of change because of its clear links with well-being (Csikszentmihalyi, 2008; Seligman, 2011). Songwriting studies by Baker and MacDonald (2013a,b) found that creating songs about positive or negative personal experiences generates strong experiences of flow in healthy populations and that participants derive meaning from both the songwriting process and the song product they created. A regression analysis determined that a predictive relationship existed between meaning and flow during songwriting experiences. More recently, Silverman et al. (under review) examined the relationship between flow, meaning, and health and well-being during songwriting interventions in a group of adults in a psychiatric unit (study 1, *N* = 54) and in adults undergoing detoxification (study 2, *N* = 170). Although these songwriting approaches were not specifically tailored to address self-concept, correlational and multiple regression analyses determined that flow and meaningfulness of songwriting were significantly correlated and that strong flow experiences were predictors of increases in hope (study 1) and readiness to change (study 2).

As strong flow experiences and meaning activated by songwriting are predictors of readiness to change and hope in adults with substance addictions and with acute psychiatric illnesses, in the current study, we aimed to explore whether songwriting activates flow and creates meaning for people with ABI and SCI, and whether these mechanisms of change correlate with changes in well-being indicators.

### Study hypotheses

Hypothesis 1: greater improvements in self-concept will be positively correlated with lower levels of anxiety, depression, and negative affect, and increased levels of satisfaction with life, sense of flourishing, and emotion regulation.

Hypothesis 2: greater improvements in self-concept and well-being will be positively correlated with stronger feelings of flow and meaningfulness of the songwriting experience.

## Materials and Methods

### Design

**Figure 1:**
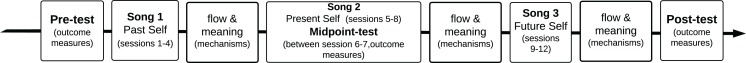
**Research design**.

A non-randomized, quasi-experimental design with repeated measures (pre–mid–post-intervention) was employed to determine: (a) whether there was a therapeutic effect (outcome measures) and (b) what mechanisms might explain this effect (mechanism measures). Outcome measures were collected at baseline, mid-point (between sessions 6 and 7), and post-intervention. Mechanisms of change (flow and meaning measures) were collected after the completion of each song during the 12-session songwriting program (see Figure 1).

The study was reviewed and approved by Human Research Ethics Committees at The University of Melbourne (1339728), Monash University (CF13/2098 – 2013001081), and Austin Health (H2013/05038).

### Participants

Over the study period, 16 inpatients with either SCI or ABI were identified as meeting the inclusion criteria and were invited to participate in the study. Inclusion criteria comprised: (1) inpatient status at Royal Talbot Rehabilitation Centre from the ABI, Spinal or Neurology wards; (2) diagnosis of SCI or ABI (including traumatic brain, stroke, brain tumor, and substance abuse); (3) aged between 18 and 65 years of age; (4) <12 months post-injury/onset; (5) cognitive capacity sufficient to complete self-report measures; (6) without significant language or hearing impairments; and (7) not in posttraumatic amnesia.

A member of each patient’s treating team (not one of the researchers) was responsible for informing the patient of the study and obtaining consent. Two patients declined to participate, one female patient was recruited but found the self-report measures too emotionally confronting and dropped out before treatment commenced, and three other participants had substantial amounts of missing data and were subsequently excluded from the analysis. Ten participants (five ABI and five SCI) completed the study; nine males and one female aged between 20 and 64 years of age (Mean 38.9, SD 13.2). The time since injury or incident ranged from 30 to 157 days (Mean 89.6, SD 44.29).

### Procedure and music therapy approach

Following recruitment, participants completed a battery of tests via iPad before engaging in a 12-session-targeted songwriting program (Tamplin et al., 2015). During the 12 (twice weekly, 1 h) sessions, the therapist and participant co-created three songs using a narrative songwriting approach (Baker, 2015). Each song incorporated the various domains of self-concept: personal, social, family, physical, academic, and moral/spiritual self. Song 1 (sessions 1–4) was focused on these domains for the past self, song 2 (sessions 5–8) was focused on these domains for the present self, and song 3 (sessions 9–12) was focused on these domains regarding the conceptualized future self. The therapist worked carefully with the participants to ensure that their stories of self were authentically represented in each song both musically and lyrically so that they could make meaning from their stories and self-descriptions. Further details of the intervention and the role of the therapist in facilitating the song creations are presented in a previously published paper (Tamplin et al., 2015).

### Outcome measures

Therapeutic outcomes were determined by collecting data pre–mid–post-intervention using the following battery of measures.

#### Self-Concept

As self-concept was the primary outcome measure of interest in this study, the 20-item *Head Injury Semantic Differential Scale* (HISDS; Tyerman and Humphrey, 1984) was used to measure self-concept. It uses adjective pairs, such as unfeeling-caring, worried-relaxed, etcetera, along a 7-point Likert Scale, to determine their view of various aspects of self. This measure focuses on perception of personal attributes with scores ranging from 20 to 140, where higher scores indicate a healthier, more positive, self-concept. The HISDS has been used previously in studies with people who have ABI (Vickery et al., 2005).

#### Well-being Measures

Well-being data were collected using seven different measures to evaluate sense of flourishing, life satisfaction, coping, affect, depression, and anxiety.

*The Flourishing Scale* is an eight-item measure of psychological well-being, specifically self-perceived success in areas such as relationships, self-esteem, purpose, and optimism. Statements are rated across a 7-point Likert Scale with scores ranging from 6 to 56; higher scores indicate stronger sense of flourishing. The measure has good psychometric properties with Cronbach’s α of 0.87, temporal stability of 0.71, and construct validity ranging from 0.43 to 0.70 (Diener et al., 2010). Mean of flourishing for healthy populations range from 42.2 (Singaporean sample, SD = 6.4) to 46.6 (New Jersey Sample SD = 5.0). The Flourishing Scale has been used previously in studies with people who have an ABI (White, 2014).

The *Satisfaction with Life Scale* (SWLS; Diener et al., 1985) is a five-item scale designed to measure satisfaction with life. The items are scored using a 7-point Likert scale with scores ranging from 5 (low satisfaction) to 35 (high satisfaction). It has good construct validity (ranging from 0.5 to 0.75), test–retest reliability (0.82–0.84), and internal consistency (0.87) (Diener et al., 1985). Normative data for adults range from 23.6–27.9 (Pavot and Diener, 1993/2009). Normative data have been collected for people at 1 and 2 years post-ABI with mean scores of 20.32 (SD = 8.13) and 20.80 (SD = 8.42), respectively, and 22.7 (SD = 7.28) for people with SCI <60 days post-injury (Fortmann et al., 2013).

*The Emotion Regulation Questionnaire* (ERQ; Gross and John, 2003) is a 10-item questionnaire designed to assess individual differences in the habitual use of two emotion regulation strategies: cognitive reappraisal (six items) and expressive suppression (four items). Items are rated on a scale from 1 (strongly disagree) to 7 (strongly agree) with scores ranging from 6 to 42 (Reappraisal) and 4 to 28 (Suppression). Mean α reliabilities were 0.79 for Reappraisal and 0.73 for Suppression and test–retest reliability across 3 months was 0.69 for both Reappraisal and Suppression scales. Higher mean scores on each subscale indicates that the reappraisal or suppression strategy is more endorsed. Testing the psychometric properties of the ERQ showed that the cognitive reappraisal subscale (α = 0.79) and expressive suppression (α = 0.73) subscales have high internal consistency for both (Gross and John, 2003). Good convergent validity has been reported with the COPE scales (Carver et al., 1989), discriminant validity with the 44-item Big Five Inventory (see Gross and John, 2003), and stability across 3 months (*r* = 0.69; Gross and John, 2003). Normative data for Reappraisal are 28.92 (6.27) and 28.48 (6.29) for females and males, respectively, and for Suppression, 13.12 (4.99) and 14.91 (4.67) for female and males, respectively (Melka et al., 2011).

The *Positive Affect and Negative Affect Scale (*PANAS-20; Watson et al., 1988) is a 20-item scale measuring the hedonic aspect of well-being using 10 positive items [Positive Affect scale (PA)], 10 negative items [Negative Affect scale (NA)]. Each item is scored on a 5-point Likert scale and each positive and negative scale ranges from scores of 10 to 50. Normative data indicate a mean score of 31.31 (SD = 7.65) and 16.00 (SD = 5.90) for the PA and NA scales, respectively (Crawford and Henry, 2004). Internal consistency of the PA was 0.89 (95% CI = 0.88–0.90) and 0.85 (95% CI = 0.84–0.87) for the NA scale. The PANAS has convergent validity when correlated with the Depression and Anxiety Stress Scale [*t*(986) = 7.523, *p* < 0.001] and the Hospital Anxiety and Depression Scale [*t*(737) = 7.667, *p* < 0.001] (Crawford and Henry, 2004). PANAS has been used in both ABI (e.g., Juengst et al., 2014) and SCI research (Salter et al., 2013).

The *Patient Health Questionnaire-9* (PHQ-9; Kroenke et al., 2001) is a nine-item scale that screens for severity of depression. Each item is scored from 0 to 3 with the total scale scores ranging from 0 to 27. Higher scores are indicative of moderately severe (15–19) and severe (20–27) levels of depression. Internal reliability (Cronbach’s α of 0.86–0.89) and convergent validity as measured against the SF-20 Health-related Quality of Life Scale (*p* < 0.05 for most pairwise comparisons) were good (Kroenke et al., 2001). The PHQ-9 has been validated on both ABI (Fann et al., 2005) and SCI samples (Bombardier et al., 2012).

The *Generalized Anxiety Disorder* scale (GAD-7; Spitzer et al., 2006) is a 7-item measure of generalized anxiety for use with the general population, measured along a 4-point Likert scale. Scores range from 0 to 21, with scores of ≥5, ≥10, and ≥15 representing mild, moderate, and severe anxiety symptom levels, respectively (Löwe et al., 2008). The internal consistency of the GAD-7 was excellent (Cronbach’s α = 0.92) and test–retest reliability was also good (intraclass correlation = 0.83). Construct validity was good as evidenced by strong association between increasing GAD-7 scores and worsening function on Short-Form Health Survey (SF-20) and convergent validity was good when compared with Beck Inventory (*r* = 0.72) (Spitzer et al., 2006).

### Mechanisms of change measures

To capture changes in flow and meaningfulness of the songwriting experience throughout the process, three measurement tools were used.

#### Short Flow Scale and Core Flow Scale

After completion of each of the three songs (after sessions 4, 8, and 12), participants completed the 9-item Short Flow Scale (SFS) and the 10-item Core Flow Scale (CFS) to measure their subjective absorption and motivation during the songwriting task (Martin and Jackson, 2008). High levels of flow are indicative that the activity is engaging and meaningful to them. While flow may have been experienced during any songwriting session, we chose to only measure flow after sessions 4, 8, and 12 to minimize assessment burden for participants.

The SFS measures the strength of the nine dimensions of flow (challenge-skill balance, action-awareness merging, clear goals, unambiguous feedback, concentration on task, sense of control, loss of self-consciousness, time transformation, and autotelic experience). When their scores are combined, they represent a measure of the psychological state of flow. The CFS, however, measures the strength of the lived experience of flow, rather than a psychological state. Both the SFS and CFS have demonstrative internal validity (CFI = 0.97; SRMR = 0.05). Construct validity has been tested across several domains (work, sport, and music) and has acceptable reliability (Cronbach’s α = 0.82; Martin and Jackson, 2008). Internal validity for SFS flow in work and sport were good, and more so for music (χ^2^ = 136.78, 112.38, and 44.11, respectively) and external validity was also good (χ2 = 6088.56, 4479.03, and 4056.76, respectively).

#### Meaningfulness of Songwriting Scale

*Meaningfulness of Songwriting Scale* is a 21-item scale constructed to measure the meaningfulness of the songwriting process and the song product post-creation Baker et al. (under review). This measure was administered to participants after the completion of each song (after sessions 4, 8, and 12). Developed by Baker and MacDonald (2013a,b), the self-report scale measures 11 domains of meaning relevant to songwriting experiences and the song product: enjoyment, discovery/self reflection, arousal of emotions, creativity, engagement, challenge, understanding context, associations, achievement, personal value, and identity. Items are measured on a 5-point Likert scale with total scores ranging from 21 to 105. Larger numbers are indicative of stronger meaning derived from the songwriting experience and song product. The measure has good face validity, strong internal consistency (Cronbach’s α 0.96), test–retest reliability (ICC = 0.89–0.93), and construct validity (*r* = 0.56–0.68) when used with people with acute mental illness and with people who have substance use disorder Baker et al. (under review). Its psychometric properties have not been measured with people with neurodisability. We have noted anecdotally that people with ABI and SCI find songwriting a meaningful experience (Baker et al., 2005d; Tamplin, 2006). The questions in the scale were intentionally worded as simply and clearly as possible to make the scale appropriate to use for people with mild cognitive impairments.

### Analyses

The data set was first screened for missing data, and occasional missing data were found for one time-point for at most two participants on any variable. Given the missing data points were not systematic in any way, analyses were conducted on the available remaining data for each variable. Distributions for all variables were generally normal and no outliers were identified, and despite the small sample sizes, parametric analyses were performed to maintain sufficient sensitivity (although caution is advised in interpreting significant findings due to the small sample sizes).

Self-concept and well-being outcome measures were operationalized as the change in each measure from baseline to mid-point (time 2–time 1), mid-point to post-intervention (time 3–time 2), or baseline to post-intervention (time 3–time 1). Flow and meaningfulness of songwriting measures were obtained by averaging ratings provided for the three songs. Pearson’s bivariate correlations (two-tailed, α set at 0.05) were performed to test the relationship between measures for each hypothesis.

## Results

### Potential confounds

As both age and time since head injury could potentially influence self-concept and well-being outcome measures independent of the songwriting intervention, Pearson’s correlations were first performed and showed no significant covariates.

The measures of self-concept and well-being at baseline, mid-intervention, and post-intervention are detailed in Table 1. The mean self-concept at baseline was 94.30 (SD = 25.88) out of a possible range 20–140, indicating a moderate view of the self-concept. Participants did not have a very poor or negative self-concept, but it was not particularly positive either. Mean self-concept improved across time and the effect size of this improvement in self-concept was medium (*d* = 0.557). Baseline levels of depression (M = 9.7, SD = 6.15) were bordering on moderate depression (10–14) and decreased to mild depression at post-intervention, and also had a medium effect (*d* = 0.682). Anxiety levels at baseline were at the lower end of the moderate anxiety range and decreased to the mild anxiety range at post-intervention. Negative affect at the baseline (M = 22.90) was higher than a normative sample (M = 16.00, Crawford and Henry, 2004) but also decreased at post-intervention, with a small effect size (*d* = 0.461). Positive affect (M = 34.5) was slightly above the normal range (M = 31.31). Baseline Satisfaction with Life Scale data were below normative levels but moved toward this over time (M = 21.78). Emotion regulation (suppression) was below the normative data set and the appraisal was higher.

**Table 1:** **Levels of self-concept and well-being across the different time-points**.

Variable (range)	Baseline			Mid-intervention			Post-intervention			*d*
	M	SD	*N*	M	SD	*N*	M	SD	*N*
Self-concept (20–140)	94.30	25.88	10	106.88	16.58	8	109.00	26.88	10	0.557*
Negative affect (10–50)	22.90	6.89	10	19.57	6.63	7	19.50	6.95	10	0.491
Depression (0–27)	9.70	6.15	10	5.75	3.20	8	5.90	4.93	10	0.682*
Anxiety (0–21)	5.10	6.06	10	4.25	3.92	8	4.56	4.90	9	0.098
Suppression emotion regulation (4–28)	12.90	6.26	10	17.63	6.65	8	12.40	4.53	10	0.092
Positive affect (10–50)	34.50	10.71	10	33.57	7.74	7	35.70	8.87	10	0.122
Flourishing (6–56)	44.33	12.56	9	47.75	5.60	8	47.80	7.19	10	0.339
Satisfaction with Life (5–35)	18.80	9.31	10	21.57	7.11	7	21.78	6.22	9	0.376
Reappraisal emotion regulation (6–42)	29.40	5.40	10	31.75	4.40	8	30.00	5.62	10	0.108

**d* reflects effect sizes for baseline to post-intervention changes only*.

*p < 0.05.

#### The Association Between Changes in Self-Concept and Well-being Across the Intervention Period

Table 2 shows means and SD for the change in self-concept and well-being measures and the correlations between changes in the self-concept measure and each of the well-being measures, at each period between time-points (baseline, mid-intervention, and post-intervention).

**Table 2:** **Descriptive statistics and Pearson’s bivariate correlations between self-concept and well-being outcomes**.

	M	SD	Correlation with self-concept		
			*r*	*p*	*n*
**CHANGE FROM BASELINE TO POST-INTERVENTION**					
Self-concept	14.70	39.27	–	–	–
Negative affect	−3.40	9.62	−0.69*	0.026	10
Depression	−3.80	7.10	−0.87*	0.001	10
Anxiety	0.67	7.60	−0.87*	0.003	9
Suppression emotion regulation	−0.50	6.79	0.25	0.479	10
Positive affect	1.20	13.90	0.73*	0.016	10
Flourishing	2.56	16.92	0.85*	0.003	9
Satisfaction with life	4.11	10.41	0.87*	0.003	9
Reappraisal emotion regulation	0.60	9.01	0.48	0.162	10
**CHANGE FROM BASELINE TO MID-INTERVENTION**					
Self-concept	16.38	26.22	–	–	–
Negative affect	−2.86	11.26	−0.96*	<0.001	7
Depression	−4.50	6.14	−0.68	0.063	8
Anxiety	−1.75	5.92	−0.77*	0.025	8
Suppression emotion regulation	6.50	6.85	0.07	0.864	8
Positive affect	−1.29	8.90	0.35	0.443	7
Flourishing	3.57	13.31	0.67	0.102	7
Satisfaction with Life	3.43	11.41	0.96*	0.001	7
Reappraisal emotion regulation	3.13	7.61	−0.15	0.715	8
**CHANGE FROM MID-INTERVENTION TO POST-INTERVENTION**					
Self-concept	6.63	13.56	–	–	–
Negative affect	−1.86	4.67	−0.09	0.856	7
Depression	−0.63	4.81	−0.88*	0.004	8
Anxiety	0.71	4.71	−0.79*	0.034	7
Suppression emotion regulation	−6.00	3.21	−0.24	0.562	8
Positive affect	3.57	10.08	0.72	0.066	7
Flourishing	2.00	5.50	0.88*	0.004	8
Satisfaction with life	0.50	2.88	−0.08	0.885	6
Reappraisal emotion regulation	−1.38	7.19	0.29	0.489	8

It can be seen from Table 2 that across the entire intervention period there were significant negative correlations between improvements in self-concept scores and decrease in ratings of negative affect, depression, and anxiety, and significant positive correlations with improvement in positive affect, flourishing, and satisfaction with life scores. From baseline to the mid-intervention point, there was a significant negative correlation between improvements in self-concept score and decreases in both negative affect and anxiety ratings, and the correlation with depression ratings approached significance. A positive correlation was also observed in change in self-concept scores over this period and satisfaction with life ratings. From mid-intervention to end of intervention, a negative correlation was observed between self-concept scores and ratings of both depression and anxiety, and a positive correlation was observed between change in self-concept score and flourishing ratings.

#### The Association Between Self-Concept and Well-being Outcome Measures and Mechanisms of Change Measures (Flow and Meaning)

Table 3 shows the means and SD for change in self-concept and well-being measures, and for mechanisms of change (flow and meaningfulness of songwriting) averaged across the entire intervention period (that is, from baseline to post-intervention), and the bivariate correlations between these variables.

**Table 3:** **Descriptive statistics for mechanisms of change variables, and correlations with change (from baseline to post-intervention) in self-concept and well-being variables**.

	M	SD	cSC	cNA	cDep	cAnx	cSupp	cPA	cFlour	cSWL	cReapp
State flow	4.02	0.40	−0.10	0.33	−0.14	0.43	−0.25	−0.40	0.06	−0.25	−0.33
			0.808	0.423	0.744	0.333	0.543	0.324	0.893	0.594	0.426
Core flow	4.14	0.46	0.02	0.02	−0.14	0.32	−0.13	−0.24	0.157	−0.23	−0.12
			0.954	0.960	0.735	0.490	0.752	0.571	0.737	0.618	0.772
Meaningfulness of songwriting	90.80	8.25	−0.39	0.68*	0.43	0.74*	−0.73*	−0.58	−0.52	−0.31	−0.57
			0.273	**0.031**	0.216	** 0.023**	** 0.018**	0.079	0.150	0.418	0.087

*cSC, change in self-concept; cNA, change in negative affect; cDep, change in depression; cAnx, change in anxiety; cSupp, change in suppression emotion regulation; cPA, change in positive affect; cFlour, change in flourishing; cSWL, change in satisfaction with life; cReapp, change in reappraisal emotion regulation*.

**N*s range from 7 to 10*.

*p < 0.05.

Table 3 reveals that the flow measures did not correlate with any of the changes in self-concept or well-being variables. The Meaningfulness of Songwriting Scale correlated positively with increased negative affect, anxiety and suppressive emotion regulation, and the negative correlation with increased positive affect approached significance.

## Discussion

### Changes to self-concept and well-being

Research indicates that integrating a past, present, and future self is difficult for people with ABI and SCI, and the self-concept does not improve naturally over time (Anson and Ponsford, 2006; Kelly et al., 2013). This study found that self-concept changed from baseline to post-intervention [mean change 14.70 (39.27)] supporting our theory that songwriting can strengthen the positive aspects of the self-concept and make a difference in how people with disability view themselves at a critical time in their rehabilitation process (Tamplin et al., 2015).

The data revealed that the largest changes in self-concept emerged from baseline to mid-point (first six sessions). During this phase of the program, the participants were exploring the past (first four sessions) and present self (sessions 5 and 6). This positive change in self-concept suggests that they had a more positive view of themselves when compared with baseline. The theory underpinning this study proposed that by exploring the past self, participants were guided to explore who they were as people prior to their injury – a focus on the residual self that may have been forgotten or hidden by the more prominent issue of the current disabled self (Lennon et al., 2014). The process of focusing on who they were prior to their injury (song about the past self), and which parts of themselves remain the same (song about the present self), led to a stronger, healthier self-concept by the mid-point assessment. As participants continued to explore the present self (sessions 7–8) and then the future self (sessions 9–12), there was a further strengthening of the self-concept although this change was less marked than during the initial six sessions. This might indicate that although there were additional improvements in self-concept that exploring the future has less benefits than reflecting on the past and the here-and-now. Perhaps for some participants, the future remains too uncertain at this early stage in their rehabilitation journey and therefore creating songs about the future self should be introduced at a later period of time post-injury. Alternately, it may be that after an initial rapid improvement in self-concept, further improvements are more gradual. In this case, greater improvements might be detected if a follow-up measurement was performed at a later stage.

Correlations between changes in self-concept and other well-being indicators were significant from baseline to post-intervention in all cases except for in the Emotion Regulation Subscales (suppressive and reappraisal). The data showed that as self-concept improved, this was positively correlated with enhanced sense of flourishing, positive affect, satisfaction of life, and was also significantly correlated with reductions in anxiety, depression, and negative affect. This indicates that as self-concept improved during the songwriting process, other well-being measures also improved. Low levels of self-concept have been associated with higher levels of depression and anxiety (Anson and Ponsford, 2006; Kelly et al., 2013). Therefore, it is noteworthy that our songwriting intervention had the reverse effect of improving self-concept and simultaneously reducing levels of anxiety and depression.

Improvements in affect, sense of flourishing, and satisfaction in life, and reductions in depression, anxiety, and negative affect are all important goals during the initial months post-injury. This is the time when recovery is most rapid and focused attention on rehabilitation is imperative (Schultz and Tate, 2013). It could be hypothesized that a process that involves grieving the past self, and facing the present and imagined future self might lead participants to overly focus on their disabilities and in doing so, negatively affect well-being. However, this was not the case in our study. The songwriting process enabled participants to be reminded of the residual self and this led to positive well-being outcomes. While other music therapy studies have not examined songwriting’s impact on affect at baseline-mid-post, other music therapy interventions addressing other rehabilitation goals have been found to facilitate an improvement in affect and mood in people with SCI (Tamplin et al., 2013a) and ABI (Baker and Wigram, 2004; Tamplin et al., 2013b).

### Mechanisms of change

It was hypothesized that strong experience of flow, and high levels of meaningfulness of the songwriting experience are mechanisms active in the songwriting process that would contribute to a change in self-concept and other well-being indicators (Tamplin et al., 2015). The findings in this study did not support the hypothesis that strong flow was associated with improved self-concept and well-being indicators. In other words, having a stronger sense of flow had no bearing on whether the participant had a greater change in self-concept or well-being when compared with participants who reported low levels of flow. The strength of meaning derived from the songwriting experience and song product did, however, significantly correlate with some well-being indicators; however, these correlations were in the opposite direction than expected. These correlations suggest that, as the songwriting experience becomes more meaningful, individuals’ levels of anxiety and negative affect increase, while suppression of emotion decreases. In trying to understand these unexpected findings, we propose that positive songwriting experiences within the context of a therapeutic relationship with a highly skilled music therapist may have enabled individuals to start accepting their emotions, which led to an increase in anxiety and negative affect. Being authentic and honest with oneself in times of stress and grief can be challenging. However, when a process such as songwriting is meaningful and enables a person with an ABI or SCI to feel safe explore aspects of their self that they might otherwise suppress, the initial effect may be positive, but as a person reflects on the content of their songs over time, it may cause negative feelings to emerge into consciousness. Our finding is not necessarily an unfavorable outcome, as it is not possible to process fears and anxieties until they are acknowledged. The music therapist has specialist skills in enabling people to explore painful issues within the safety of a therapeutic relationship and within the safety of musical experiences so that these fears and anxieties can be addressed.

### Overall findings

When considering the two hypotheses of this study, there seems to be some contradictory findings. First, songwriting positively affected self-concept over time and this was, as hypothesized, correlated with positive changes in well-being. However, higher states of flow and more meaning derived from the songwriting experience were not significantly correlated with positive changes to self-concept and well-being, and at times, the trends were in the opposite direction than predicted. One explanation for why our second hypothesis was rejected stems from the possibility that participants were completing flow scales that had not previously been psychometrically tested with this population. It is unclear whether participants were able to reflect on their experiences well enough to be able to rate their experience of flow. The same may be said for the Meaningfulness of Songwriting Scale, which has to date only been psychometrically tested in a mental health population Baker et al. (under review). Hence, it is possible that this measure may not have accurately captured the meaningfulness of the experience for our study’s populations. Further, it is possible that the timing of the flow measures affected the results. If flow had been measured after each of the sessions rather than at the completion of each song, stronger flow experiences during the lyric writing or music creation process may have been identified. This would have provided a deeper understanding of whether flow is stronger at different points during the songwriting process.

An alternative explanation for the absence of positive correlations between the mechanisms of change (flow and meaning) and self-concept and well-being could be that other mechanisms are more significant contributors to a change in self-concept and well-being. Given that the songwriting protocol systematically facilitates an exploration of the full range of self-concept domains (physical, personal, family, social, moral, and academic self), perhaps this self-exploration and narrative approach (that just so happens to incorporate a songwriting experience), is the critical, mediating factor that enables the multiple aspects of self to be more integrated (Feinstein and Krippner, 2008). Similarly, the role of the therapist in offering support when the participant grieves lost parts of the self, challenging a participant’s self view, or presenting potential alternative perspectives (Obodaru, 2012), might have a strong impact on changes to self-concept and well-being over time. If this is the mediating mechanism of change, the role of songwriting is therefore to provide a supportive yet challenging and stimulating context in which the narrative experience may evolve. Songs are an age-appropriate and culturally accepted medium for communicating people’s stories (Baker, 2015). They provide a framework where key events, feelings, or self-perspectives can be highlighted in a chorus, thereby encouraging further processing, and more effective consolidation into memory (Cahill and McGaugh, 1996; Judde and Rickard, 2010). Finally, our findings indicated a positive change in mood and emotional well-being across the 12 sessions, supporting our earlier proposed ideas (Tamplin et al., 2015) that songwriting – a music-based intervention – engages the mesolimbic system in the brain and in doing so affects mood, depression, anxiety, and coping (Menon and Levitin, 2005; Salimpoor and Zatorre, 2013).

The proposed theory that (a) music facilitates consolidation of the self-exploration process into memory and (b) the role of the narrative process is pivotal in addressing self-concept that deserves further investigation. A study that compares the effects of narrative therapy with narrative songwriting on self-concept and well-being with cognitively compromised people (issues of ongoing memory) may shed light into the role of the songwriting process in reconstructing the self post-injury.

### Limitations of the study

This study comprised a small sample size of two cohorts (ABI and SCI) whose data were pooled. The sample size was insufficient to allow separate examination of the cohorts. Larger sample sizes would enable population differences to emerge regarding the effects of songwriting on self-concept and well-being, as well as the mechanisms of change. While measures for some outcome variables have been psychometrically tested, or at least been used, in other studies with people who have ABI or SCI (HISDS, SWLS, Satisfaction with Life Scale, Flourishing Scale, PANAS, and PHQ-9), the ERQ and GAD-7 have not. It is unclear whether these scales are valid for use with people who have SCI and ABI. Similarly, the Meaningfulness of Songwriting Scale is a newly developed scale and only has data on two samples (patients in detoxification for substance use disorder and acute psychiatric patients). Finally, given the small sample sizes, non-parametric analyses may have been more cautious although would have reduced power considerably and therefore parametric analyses were retained. However, the significant findings should be interpreted as preliminary in nature and require replication with a larger sample size.

This study did not have a comparative or control condition to determine whether the changes in self-concept and well-being were due to natural recovery or were indeed an outcome of the songwriting intervention. As self-concept has not been found to improve naturally over time (Kelly et al., 2013), we have made an assumption that the songwriting intervention effected this change; however, this cannot be confirmed until a larger study with sufficient power has been implemented using a comparative or control condition with random assignment. Further, it is possible that the songwriting program was a distraction from thinking about their losses and thus led to the positive change in well-being. This is unlikely, however, because the program was directing participants to reflect on the self rather than distract them from thinking about the self and their future.

It is likely that strong flow experiences were evident across each of the 12 sessions of songwriting. Measuring flow after each of the 12 songwriting sessions may have yielded more data about how flow was experienced over the whole songwriting process. Such data may have enabled stronger correlations between the flow experiences and changes in self-concept and well-being measures to be captured. It is therefore recommended in future research that flow is measured after each songwriting session to provide a more complete picture of the experience of flow throughout the songwriting process.

## Conclusion

This study has examined the impact of a therapeutic songwriting program on the self-concept and well-being of people with ABI and SCI, with a specific focus on measuring hypothesized mechanisms of change. Our songwriting protocol was specifically designed to explore the various domains of the self-concept via the creation of three songs about the past self, present self, and future self. We found that changes to self-concept and well-being facilitated by the intervention were highly correlated and changed in a positive direction indicating that people currently undergoing rehabilitation for SCI or ABI benefit from such a strategic songwriting approach. There were no correlations between levels of flow and self-concept and other well-being measures but found correlations with meaningfulness in the inverse-to-hypothesized direction. In particular, as the strength of the meaningfulness of the songwriting experience increased, levels of anxiety and negative affect increased and emotional suppression decreased. We propose that there may be other mechanisms more critical in facilitating the positive changes in self-concept and well-being that emerged in this study, such as the role of story-telling and the impact of music in facilitating the consolidation of self-concept explorations in memory.

## Conflict of Interest Statement

The authors declare that the research was conducted in the absence of any commercial or financial relationships that could be construed as a potential conflict of interest.
